# Why do seals have cones? Behavioural evidence for colour-blindness in harbour seals

**DOI:** 10.1007/s10071-014-0823-3

**Published:** 2014-12-02

**Authors:** Christine Scholtyssek, Almut Kelber, Guido Dehnhardt

**Affiliations:** 1 Lund Vision Group, Functional Zoology, Department of Biology, Lund University, Sölvegatan 35, 22362 Lund, Sweden; 2 Sensory and Cognitive Ecology, Institute for Biosciences, Rostock University, Albert-Einstein-Str. 3, 18059 Rostock, Germany

**Keywords:** Rod–cone-based colour vision, Colour-blindness, Spectral sensitivity, Marine mammals, Harbour seals

## Abstract

All seals and cetaceans have lost at least one of two ancestral cone classes and should therefore be colour-blind. Nevertheless, earlier studies showed that these marine mammals can discriminate colours and a colour vision mechanism has been proposed which contrasts signals from cones and rods. However, these earlier studies underestimated the brightness discrimination abilities of these animals, so that they could have discriminated colours using brightness only. Using a psychophysical discrimination experiment, we showed that a harbour seal can solve a colour discrimination task by means of brightness discrimination alone. Performing a series of experiments in which two harbour seals had to discriminate the brightness of colours, we also found strong evidence for purely scotopic (rod-based) vision at light levels that lead to mesopic (rod–cone-based) vision in other mammals. This finding speaks against rod–cone-based colour vision in harbour seals. To test for colour-blindness, we used a cognitive approach involving a harbour seal trained to use a concept of same and different. We tested this seal with pairs of isoluminant stimuli that were either same or different in colour. If the seal had perceived colour, it would have responded to colour differences between stimuli. However, the seal responded with “same”, providing strong evidence for colour-blindness.

**Electronic supplementary material:**

The online version of this article (doi:10.1007/s10071-014-0823-3) contains supplementary material, which is available to authorized users.

## Introduction

Seeing the world in colour allows for reliable object detection and recognition under variable illumination. For an animal to see colour, its retina has to contain at least two spectrally distinct photoreceptor types whose signals are compared in colour opponent mechanisms (Kelber et al. 2003). Most terrestrial mammals possess two spectral classes of cones, SWS1 (short-wavelength-sensitive) and LWS (long-wavelength-sensitive) (Jacobs 2009; Kelber et al. 2003; Peichl 2005). A few nocturnal species lack SWS1 cones and are therefore LWS monochromats (Deegan and Jacobs 1996; Jacobs 2013; Jacobs et al. 1996; Peichl and Moutairou 1998; Peichl and Pohl 2000). What seems to be an exception among terrestrial mammals has evolved to be the rule in the two largest groups of marine mammals. All cetaceans and seals that have been investigated have lost their SWS1 cones and hence the basis of cone-based colour vision (Crognale et al. 1998; Fasick et al. 1998; Levenson and Dizon 2003; Levenson et al. 2006; Newman and Robinson 2005; Peichl and Moutairou 1998). Some species of whales [Balaenidae, Balaenopteroidea, the Sowerby’s beaked whale (*Mesoplodon bidens*), the giant sperm whale (*Physeter macrocephalus*), and the pygmy sperm whale (*Kogia breviceps*)] have even lost both cone types and are therefore rod monochromats (Meredith et al. 2013). These findings suggest that a secondarily aquatic lifestyle (in contrast to a terrestrial lifestyle) favours colour-blindness. The reason could be the narrow spectra of light that whales and seals encounter when foraging in coastal waters or at greater depth (Jerlov 1951). If the spectral bandwidth is too small to ensure colour constancy, benefits of colour vision—such as facilitated object detection and recognition—are lost. Furthermore, colour vision compromises sensitivity, and considering the small amount of light that is left for most whales or seals during foraging, colour vision may have been lost in favour of the absolute sensitivity of the visual system.

Surprisingly, early behavioural investigations seem to have demonstrated that marine mammals see colour. Wartzok and McCormick (1978) showed that one of two Bering Sea spotted seals (*Phoca largha*) discriminated blue from orange light. Other behavioural studies investigated colour discrimination in South African fur seals (*Arctocephalus pusillus*), South American fur seals (*Arctocephalus australis*), California sea lions (*Zalophus californianus*), and a bottlenose dolphin (*Tursiops truncatus*) (Busch and Dücker 1987; Griebel and Schmid 1992, 2002). The general conclusion of all studies was that these cone monochromats see colour, and the hypothesis arose that marine mammals may have colour vision mediated by an opponent mechanism contrasting neural signals from LWS cones and rods (Crognale et al. 1998). However, those studies underestimated the sensitivity for brightness differences in these animals (Scholtyssek and Dehnhardt 2013; Scholtyssek et al. 2008) and therefore did not control sufficiently for the relative brightness of the stimuli that the animals were trained to discriminate. Hence, it cannot be excluded that the demonstration of colour discrimination in previous studies was based on brightness discrimination rather than colour vision. Furthermore, for a rod–cone-based colour vision mechanism, rods and cones need to be active at the same time (mesopic vision). Flicker photometric electroretinograms (ERGs) in the California sea lion and the harbour seal failed to find any contribution of the LWS cones to the spectral sensitivity of the eye (Crognale et al. 1998; Levenson et al. 2006). Instead, the spectral sensitivity functions resembled those of the rods even at an ambient luminance of 495 lx, which leads to photopic vision in humans (van Hateren and Snippe 2007).

The goal of our study was to shed light on the paradox of anatomical and physiological findings that suggest colour-blindness and behavioural experiments that suggest colour vision in marine mammals by performing a series of psychophysical experiments with harbour seals. In Experiment 1, we used a classical approach to test for colour vision. We trained a harbour seal to discriminate between stimuli that appear blue or green to humans. The brightness of blue and green was chosen in such a way that we could determine whether the seal used brightness or colour to solve the discrimination task. Since the contrasts between blue and green differ for scotopic (rod-based) and photopic (cone-based) vision, we could also determine which photoreceptors mediated brightness perception in the harbour seal.

With a classical approach like the one used in Experiment 1, it is hard to prove colour-blindness, since the animal could have failed to learn the discrimination but still see colour. To overcome this problem, we used a cognitive approach to test for colour vision in Experiment 2, which involved a harbour seal that had learned to form a concept of sameness and difference in a previous study (Scholtyssek et al. 2013). That study demonstrated that the seal could use this concept to judge whether completely unfamiliar stimuli were same or different irrespective of the dimension in which they differed (shape, brightness, or pattern). In the present study, we confronted the harbour seal with stimuli that differed only in colour (blue vs. green) and tested whether it would perceive them as “same” or as “different”. This way we found convincing evidence for colour-blindness.

## Experiment 1: discrimination of green and blue

### Materials and methods

#### Experimental animal

The experimental animal was a 12-year-old male harbour seal named Nick. He was housed with eight conspecifics and one fur seal in the open-sea enclosure of the Marine Science Center in Rostock, Germany. Nick was experienced in learning and performing visual discriminations.

#### Apparatus and stimuli

To ensure a constant state of adaptation, all experiments were conducted in a light-tight experimental chamber (2 m wide, 3 m long, and 2.2 m high). On command of the experimenter, the seal could enter the chamber through a sliding door (a picture of the chamber can be found in Scholtyssek et al. 2013). Illumination was provided by white LEDs (Conrad, Telux LED TLWW 7600; spectral bandwidth: 400–800 nm) powered by an adjustable constant current source (Voltcraft, type 3610) that produced a well-controlled and evenly distributed illumination of 0.9 lx in the area surrounding the experimental apparatus. This is equivalent to a luminance of 0.5 cd/m^2^ (measured with a Minolta luminance meter). This luminance corresponds to the lower range of mesopic vision in mammals, including humans, whose mesopic range falls between 0.001 and 10 cd/m^2^ (Hammod and James 1971; Virsu et al. 1987).

To avoid giving unintentional cues, a black polyethylene screen was installed in the chamber that separated the animal from the experimenter who could observe the animal via a mirror outside the animal’s field of view. A 17″ TFT monitor (Eizo FlexScan) for stimulus presentation was placed behind a window in the screen (5.5 cm from the floor). A stationing target was placed on the floor 25 cm from the centre of the monitor. Two response targets were placed directly in front of the screen beneath the stimuli that were presented on the monitor. A diagram of the experimental apparatus can be found in online resource 1.

**Fig. 1:**
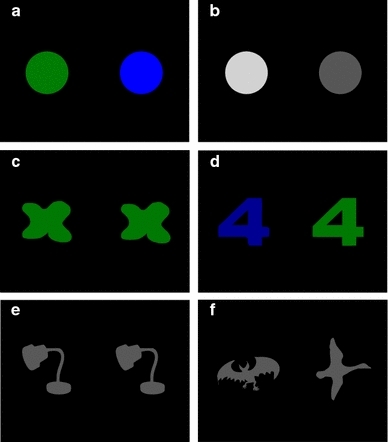
Examples of the pairs used in the three different experiments. **a** Blue–green pair used in Experiment 1 and 2. **b** Grey pair used in Experiment 1. **c** Colour “same” pair, **d** colour “different” pair, **e** shape “same” pair, and **f** shape “different” pair used in Experiment 3. The dimensions of the stimuli are described in the methods section of each experiment

Stimulus pairs consisted of a blue and a green disc of different intensities presented on a black background (Fig. 1a). Each disc comprised a visual angle of 12.6°.

The two discs had a distance of 32° between their centres, as seen from the stationing target. Different intensities of green and blue were generated using different values of either the green (G) or the blue (B) channel of the graphics card in Microsoft PowerPoint.

Eighteen blue–green pairs were used in the experiment. To make brightness an unreliable cue for the seal’s choice, blue was brighter than green in nine pairs and darker than green in the other nine pairs. Brightness (*Q*_i_) was calculated as quantum catch by weighting the spectral irradiance (*I*_i_) with the spectral sensitivity function (*S*) of either the rods (*λ*_max_ 496 nm as found by Lavigne and Ronald 1975) or the LWS cones (*λ*_max_ 553 nm as found by Levenson et al. 2006) using Eq. 1 (Vorobyev and Osorio 1998).

1 $$Q_{i} = \int\limits_{300}^{700} {I_{i} } \left( \lambda \right) \cdot S\left( \lambda \right) \cdot d\left( \lambda \right)$$

The spectral irradiance of the stimuli was measured with a USB2000 spectrophotometer (Ocean Optics Germany GmbH). Spectral sensitivity was modelled using the template of Govardovskii et al. (2000).

Brightness contrast (*C*) between blue and green for rods or cones was calculated following Eq. 2:

2 $$C = \frac{{\Delta Q}}{{Q_{\hbox{max} } }},$$

where Δ*Q* is the brightness difference between blue and green and *Q*_max_ is the brightness of the brighter colour. Since rods are generally more sensitive to blue light, and LWS cones are more sensitive to green light, the brightness contrasts between blue and green in the present experiment differed for scotopic and photopic vision. As a result, a pair of bright blue and dark green has a high contrast for the rod and a low contrast for the cone, and the opposite is true for dark blue and bright green. Based on this, two sets of blue–green stimulus pairs were used: in *Set 1*, the brightness contrast between blue and green was generally above the threshold of 14 % (Scholtyssek et al. 2008), but in pairs in which blue was brighter than green, contrast was generally higher for the rods (with the exception of stimulus pair 5, Table 1) and in pairs in which blue was darker than green, the contrast was higher for the cones. In *Set 2*, the brightness contrast for rods was high for all stimulus pairs. For the cones, however, the contrast was low when blue was brighter than green (Table 1).

**Table 1:** Rod- and cone-specific brightness contrasts *C* for the blue and green stimuli in* Sets 1* and *2*

	Stimulus pair	C rods	C cones
*Set 1*			
Blue darker	1	0.40	1
	2	0.30	0.93
	3	0.20	0.81
	4	0.20	1
Blue brighter	5	0.98	1
	6	0.85	0.38
	7	0.79	0.19
	8	0.93	0.76
*Set 2*			
Blue darker	1	0.72	0.93
	2	0.72	0.92
	3	0.69	0.91
	4	0.72	0.94
	5	0.72	0.92
Blue brighter	6	0.72	0.05
	7	0.68	0.15
	8	0.73	0.20
	9	0.72	0
	10	0.71	0.17

Contrasts are given as Weber fractions (Eq. 2)

For control experiments, we used two sets of grey stimuli whose brightness contrasts resembled the scotopic contrasts of the blue–green pairs in *Sets 1* and *2* (Fig. 1b). *Set 3* consisted of a single pair of grey discs with the same scotopic brightness contrast as the blue–green pairs in *Set 2* (70 %). *Set 4* consisted of eight pairs of grey that resembled the scotopic brightness contrasts of the stimuli in *Set 1*.

The luminance of all stimuli ranged from 0.05 to 13 cd/m^2^, which is above the luminance threshold of colour vision in mammals (Roth et al. 2008).

#### Procedure

The seal was given 5 min to adapt to the ambient luminance. A previous study on the brightness discrimination ability of the harbour seal showed that 5 min is sufficient for the harbour seal to adapt to the ambient illumination in the experimental chamber, hence ensuring a reproducible performance for a variety of stimulus brightness (Scholtyssek et al. 2008). Prior to a trial, the seal stationed in front of the monitor by touching the stationing target with its muzzle. A trial started with the presentation of a stimulus pair. To obtain a food reward, the seal had to indicate the position of the blue stimulus by touching the response target beneath it with its muzzle. A correct choice was followed by reinforcement with herring and the presentation of the black background.

Colour vision training started with the first set of blue–green pairs (*Set 1*, Table 1) presented four times in each session (32 trials). After 37 sessions, *Set 1* was substituted by *Set 2* and a session comprised 40 trials, with each pair being presented four times. During the entire experiment, stimuli were presented in pseudo-random order, while no stimulus pair was presented more than twice in a row.

After colour vision tests, grey pairs (*Sets 3* and *4*) were introduced. One session was run with *Set 3* and five sessions were run with *Set 4*. In these tests, response to the brighter stimulus was rewarded. During the entire experiment, the position of the positive stimulus was pseudo-randomized (Gellermann 1933).

For every session, the correct choices of blue were scored separately for pairs in which blue was the brighter stimulus and for pairs in which blue was the darker stimulus.

### Results

**Fig. 2:**
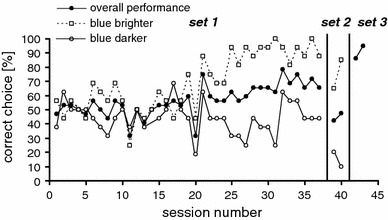
Learning curve for the three sets of stimuli used in the colour vision training and the control experiments with the seal Nick. Performance is analysed separately for trials in which the positive blue stimulus was brighter or darker than the negative green stimulus

Figure 2 shows the learning curve for the blue–green discrimination training. The seal’s performance remained at chance level for 20 sessions. In the following sessions, the performance improved and differed significantly from chance in sessions 31–37 (mean performance 70.8 % correct, Chi-square test, *n* = 192, *p* < 0.001; filled symbols in Fig. 2). This performance could be interpreted as colour vision. However, for those pairs in which blue was darker than green, the performance remained at chance level (51 % correct; *n* = 96; open squares in Fig. 2). For pairs in which blue was brighter than green, 90 % of the seal’s choices were correct (Chi-square test, *n* = 96; *p* < 0.01; open circles in Fig. 2). Apparently, the seal learned to choose the brighter stimulus instead of colour and it perceived brightness with the rods, since for scotopic vision, the brightness contrast between blue and green was much higher when blue was brighter than green (Table 1). This was when the seal could discriminate the stimuli. We tested this hypothesis with *Set 2*, in which *all* blue–green pairs had high contrast for rods. Immediately, the performance in choosing the blue stimulus dropped to chance level (filled symbols in Fig. 2) because the seal chose the brighter colour in *all* stimulus pairs, confirming that it had learned to use brightness instead of colour to make a choice.

**Fig. 3:**
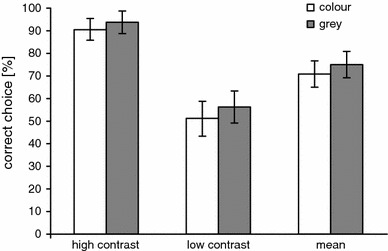
Comparison of the performance in choosing the brighter stimulus for the last five sessions (192 trials) with blue–green pairs in *Set 1* and the *grey* pairs in *Set 4* that had the same brightness contrast as the stimuli in *Set 1* for the rods. The error* bars* indicate the SD

When we introduced grey pairs (*Set 3*, Fig. 2 and *Set 4*, Fig. 3) with the same scotopic brightness contrast as in *Set 1* and *Set 2*, we found that the seal’s performance in choosing the brighter stimulus was similar to the brightness-mediated performance with the colour pairs (Figs. 2, 3). This strongly suggests that the seal perceived brightness with the rods and not with the cones.

## Experiment 2: perception of colour differences

In the first experiment, the harbour seal did not learn to discriminate blue from green, indicating colour-blindness. However, it is possible that the seal perceived colour, but ignored this information. Therefore, we tested whether a second harbour seal could respond to the colour difference between a blue and a green stimulus. In previous experiments, this seal had learned to form a concept of same and different. It could use this concept to indicate whether two completely unfamiliar visual stimuli were the same or different, irrespective of the dimension in which they differed (shape, brightness, pattern) (Scholtyssek et al. 2013). In the present study, we asked whether this seal perceived equally bright blue and green stimuli as “same” or as “different”.

For this purpose, we first determined equally bright blue and green in a series of brightness discrimination experiments with this seal.

### Materials and methods

#### Experimental animal

The experimental animal was Luca, a 9-year-old male harbour seal housed in the same facility as Nick. Luca was experienced in performing brightness discriminations and had formed the concept of same/different in previous experiments (Scholtyssek et al. 2013, 2008).

#### Apparatus

The apparatus was the same as in Experiment 1, but the stationing and response targets were substituted by a jaw station that consisted of a metal hoop fitting the girth of the seal’s head and a steel plate serving as a chin rest. The station was fixed to the floor, 50 cm from the centre of the monitor. For determining equally bright colours, two response targets were attached to either side of the jaw station. For the colour vision test, the left response target was removed. Figures of the experimental apparatus can be found in online resource 1.

#### Stimuli

1. Matching brightness of colour
  Pairs of a green and a blue, a blue and a grey, or a green and a grey disc of different intensities were presented on the monitor on a black background (Fig. 1a). Seen from the station, discs subtended a visual angle of 6.3° each and were separated by 16° (centre to centre). For the behavioural experiments, we needed to know which intensities of blue and green would be brighter or darker than grey for both rods and cones. Therefore, we calculated the quantum catches of different intensities of blue, green, and grey for rods (*λ*_max_ 496 nm), for *λ*_max_ 510 nm [as observed by Crognale et al. (1998)] and for cones (*λ*_max_ 553 nm) using the same methods as described for Experiment 1.
  We also calculated which intensities of blue, green, and grey were equally bright for rods or cones. Brightness contrasts <5 % were defined as being equally bright to the seal. This contrast is below the harbour seal’s brightness discrimination threshold of 14 % (Scholtyssek et al. 2008). The calculated brightness matches were compared to the brightness matches obtained in the brightness discrimination experiments.
2. Colour vision test
  For colour trials, 100 unique pairs of shapes (Fig. 1c, d) were filled with the green and the blue that were identical in brightness (“Matching brightness of colour” section). On average, the shapes comprised a visual angle of 13.5° the station and were separated by 14° (centre to centre). Fifty pairs had stimuli of the same colour, whereas in the other 50 pairs, green and blue stimuli were combined. As a control, we used shape trials with 100 pairs of familiar same or different shapes filled with the standard grey used in the brightness matching experiment (“Introduction” section, Fig. 1e, f).

#### Procedure

1. Matching the brightness of colour
  Prior to each session, the harbour seal was given 5 min to adapt to the ambient light. At the beginning of a session, the seal stationed in front of the monitor by placing its head in the hoop station. A trial started with the presentation of a stimulus pair. The seal indicated the position of the brighter stimulus by pulling its head from the hoop station and touching the corresponding response target with its muzzle. A correct response was reinforced with herring or sprat. After the seal made its choice, the black background was presented. A new trial started when the seal took up the position in the station again.
  To determine an exact brightness match of blue and green, both colours were first matched to the brightness of the same shade of grey. We used 14 intensities of blue and green, calculated to be brighter or darker than this standard grey. In each session, all blue or green intensities were tested against the standard grey four times, in a pseudo-random order (56 trials/session). Neither colour nor grey was the positive (brighter) stimulus for more than three consecutive trials. Each standard–comparison pair was tested 50 times. Equal brightness of colour and grey was defined as the intensity of green or blue at which the seal performed 50 % correct “brighter” responses. This value was interpolated from psychometric functions, that is, the performance in correct brighter responses plotted as a function of the intensity of the comparison stimulus.
  The intensities of blue and green that were determined to be as bright as the standard grey were chosen as standards in two additional brightness matching tests. The standard green was tested against 12 intensities of blue, and the standard blue was tested against 12 intensities of green in the same way as described above.
2. Colour vision test
  Luca had learned the same/different task used for the colour vision tests in previous experiments on same/different concept formation (Scholtyssek et al. 2013). A session started after 5-min adaptation as described above. A trial started with the presentation of one stimulus on the left side of the monitor. After 5 s, a second stimulus appeared on the right side. The seal responded with “same” by touching the response target with its muzzle within 5 s or with “different” by remaining at the station for 5 s. A correct “same” or “different” response was rewarded with herring or sprat.
  Ten sessions were performed. In each session, five colour “same” trials (blue–blue or green–green) and five colour “different” trials (blue–green or green–blue) were presented together with ten grey shape “same” and ten grey shape “different” trials. The blue and the green stimuli were presented equally often on the right and the left side. The sequence of “same” and “different” trials and the sequence of colour and shape trials were pseudo-randomized within a session, and neither colour nor shape was presented for more than three consecutive trials.

### Results

1. Matching the brightness of colour
  The intensities of green, blue, and grey that were calculated to be equally bright for a spectral sensitivity with *λ*_max_ 496 nm (scotopic), *λ*_max_ 510 nm, or *λ*_max_ 553 nm (photopic) are shown in Table 2.

**Table 2:** Comparison of the intensities of blue, green, and grey that were calculated to be isoluminant for scotopic vision (*λ*_max_ 496 nm), photopic vision (*λ*_max_ 553 nm), and an intermediate *λ*_max_ 510 nm, with the experimentally obtained isoluminant intensities

*λ*_max_	Grey versus blue	Grey versus green	Blue versus green	Green versus blue
496	1.55E+14	2.00E+14	**2.24E+14**	**1.28E+14**
510	**1.99E+14**	**1.60E+14**	**1.50E+14**	**1.90E+14**
552	4.21E+14	1.50E+14	–	4.36E+14
Match	**1.80E+14**	**1.60E+14**	**1.83E+14**	**1.60E+14**

The intensities are given as photons cm^−2^ s^−1^. Best matches between hypothetical and experimentally obtained data are indicated in bold
  The results of the behavioural brightness matches between green and grey, blue and grey, green and blue as well as blue and green are plotted as psychometric functions in Fig. 4. The shapes of the psychometric functions are best described by Boltzmann functions (Vriens et al. 2011) with *r* ^2^ ranging from 0.97 to 1. The arrows indicate the comparison intensities that were indistinguishable from the standard intensity (50 % correct brighter responses). In the tests with blue and green stimuli (Fig. 4c, d), the seal perceived the same intensities of blue and green as equally bright that it also perceived as equally bright as the standard grey (Fig. 4a, b). The results of the brightness matches obtained in the behavioural experiment are in agreement with the assumption that the spectral sensitivity of the visual system has a* λ*_max_ between 496 nm (scotopic) and 510 nm (Table 2). This confirms the finding of Experiment 1 that vision is scotopic in the harbour seal at light levels that are mesopic for humans (Hammod and James 1971; Virsu et al. 1987).

**Fig. 4:**
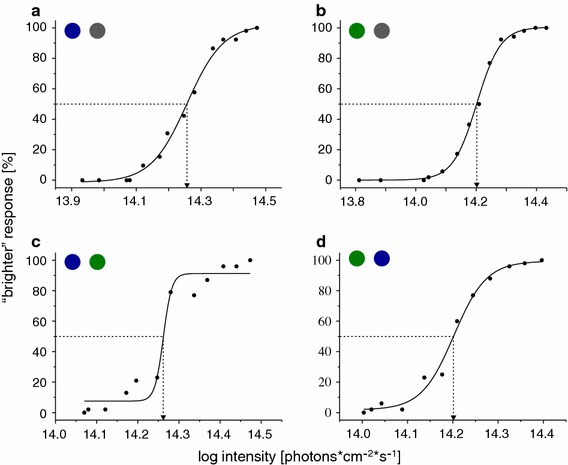
Psychometric functions of the four brightness matching tests. **a** Standard grey tested against blue. **b** Standard grey versus green. **c** Standard green versus blue and **d** standard blue versus green. The *solid lines* describe the best fit for the function. Correlation coefficients for the Boltzmann fits: **a** *r* ^2^ = 0.99, **b** *r* ^2^ = 1, **c** *r* ^2^ 0.9, **d** *r* ^2^ = 0.98. The intensities that match the standards in subjective brightness (50 % correct brighter response) are indicated by *arrows*
2. Colour vision test
  Figure 5 shows the seal’s performance on shape and colour “same” and “different” trials. The overall performance on shape trials was highly significant (80 % correct, Chi-square test, *n* = 100, *p* < 0.001) with 76 % correct “same” responses (*n* = 50, *p* < 0.001) and 84 % correct “different” responses (*n* = 50, *p* < 0.001). The performance does not differ significantly between same and different trials (Chi-square test, *p* = 0.54). The performance in shape control trials demonstrates that the seal responded on the basis of sameness and difference throughout the experiment.

**Fig. 5:**
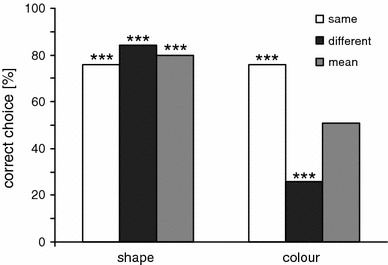
Performance of the seal Luca on shape “same” and “different” trials and colour “same” and “different” trials. The *asterisks* indicate performance above change level: ***p < 0.001
  In contrast to the shape trials, the mean performance in colour trials was at chance level (Chi-square test, 50 % correct, *n* = 100). The seal responded with “same” when both stimuli had the same colour (Chi-square test, 76 % correct choices, *p* < 0.001), but also in 74 % of the trials with one blue and one green stimulus (Chi-square test, *p* < 0.001), thus making only 26 % correct choices in the “different” trials. The proportion of correct responses to colour differences (26 %) was comparable to the proportion of incorrect responses to stimuli with identical colour (24 %). These results clearly show that the harbour seal treated stimuli that solely differed in colour as “same”.

## Discussion

### Harbour seals are colour-blind

We used two types of behavioural experiments to test for colour vision in the harbour seal while carefully controlling the relative brightness of the colours. This was especially challenging since we did not know the maximum spectral sensitivity of the harbour seal visual system, especially for mesopic light levels. In Experiment 1, we used a standard two-choice task in which one seal was reinforced for choosing a blue stimulus, whereas no reinforcement followed the choice of the green stimulus. Brightness was varied independently from colour, but the brightness contrast between blue and green was very distinct for scotopic and photopic vision. In this experiment, the harbour seal chose the blue stimulus more often than predicted by chance. Without knowledge about the spectral sensitivity of the seal, and thus the relative brightness of blue and green for the animal, one could interpret this result as proof for colour vision. However, the performance was only significant because the seal could use the brightness of the stimuli (perceived by the rods) as a cue. When the contrast between blue and green was high for scotopic vision, the seal chose the brighter stimulus (blue, dashed line in Fig. 2), but when the contrast was low, the seal chose randomly either blue or green. The fact that the seal learned to choose the brighter stimulus is proven by a chance level performance with new stimuli in *Set 2*. The brightness contrast between blue and green in these new stimulus pairs was always high for the rods, and since the seal had learned to choose the brighter stimulus, its performance dropped to chance level. This demonstrates how easily an animal can perform colour discriminations just using brightness differences if the relative brightness of the stimuli is unknown.

Although this seal did not learn the colour discrimination, it could be argued that it still perceived the colour, but ignored this information because brightness differences might have been more salient for the seal. For this reason, we used a new experimental approach with a second harbour seal, Luca, in Experiment 2. We used this seal’s ability to compare two stimuli and respond if they are “same” or “different”. In a previous study, we showed that Luca spontaneously transferred this concept of same/different to untrained visual dimensions of a stimulus (Scholtyssek et al. 2013). Hence, if Luca perceived colour, we expected him to treat two stimuli that solely differ in colour as “different”. We carefully eliminated brightness differences between the colours and showed that the seal perceived stimuli that only differed in colour as “same”. This final experiment clearly demonstrates colour-blindness.

### Colour vision in marine mammals?

The results of the present study contradict those obtained in earlier studies on marine mammal colour vision. However, in most former studies, positive results can be explained by insufficient control of the relative brightness of the stimuli. The fur seals and the California sea lions, for instance, were trained to discriminate a single colour against a series of grey shades (Busch and Dücker 1987; Griebel and Schmid 1992, 2002). The authors hypothesized that at least one of these grey shades would be confused with the brightness of the colour. However, fur seals and harbour seals are able to perceive fine brightness differences (Scholtyssek and Dehnhardt 2013; Scholtyssek et al. 2008), and if the California sea lion has equally good brightness discrimination abilities, it is likely that all species discriminated the colour from all shades of grey using brightness as the relevant cue.

In the study with the bottlenose dolphin (Griebel and Schmid 2002), brightness matches of monochromatic stimuli were calculated from a previously determined increment threshold spectral sensitivity function. In the colour vision training, the intensity of monochromatic stimuli was varied around the point of equal brightness to eliminate brightness cues. However, these experiments were performed outdoors, so that the intensity and spectral composition of the ambient light must have varied considerably, influencing the relative brightness of the monochromatic stimuli. The assumption that animals in previous studies used brightness and not colour is supported by our results obtained from the harbour seal Nick (Fig. 2) that demonstrate how an animal can succeed in a colour discrimination task using brightness instead of colour.

Another indication for a misinterpretation of the results in former studies on colour vision in marine mammals is the fact that the animals could not discriminate all colours from grey. No fur seal learned to discriminate yellow or red from grey (Busch and Dücker 1987). All three sea lions tested distinguished blue from grey, but only two individuals distinguished green from grey and no sea lion could distinguish red from grey (Griebel and Schmid 1992). The failure to discriminate some colour from grey is typical for dichromats. In dichromats, these colours are situated at the “neutral point” of their colour space, as they stimulate both receptor classes (SWS1 and LWS) to the same extent as a neutral grey. For a rod–cone dichromat, the neutral point should be at 525 nm, thus in the green range, so that it can be expected that some shades of green may be undistinguishable grey. Red, however, should stimulate the LWS cones to a greater extend than the rods and therefore should be distinguishable from grey unless the subjects were colour-blind.

Since our study contradicts results from earlier studies on colour vision in marine mammals, and since some of the studies have been conducted before it was discovered that whales and seals are monochromats, it is worth retesting these species.

A recent study has shown that some whales have also lost their LWS cones and became colour-blind rod monochromats (Meredith et al. 2013). Given that colour must be unreliable information for marine mammals that experience very low light intensities and extremely narrow light spectra during foraging, and knowing that colour vision comes at the cost of absolute sensitivity of the visual system, it would not be surprising if all whales and seals were colour-blind.

### Spectral sensitivity of the harbour seal under mesopic conditions

A big problem we faced when planning the colour vision tests was to control the brightness of the colours without actually knowing whether cones contribute to the sensitivity of the visual system. For this reason, we calculated the relative brightness of blue and green assuming different spectral sensitivities (*λ*_max_ 496 nm for rods and *λ*_max_ 553 nm for cones). We then tested the harbour seal’s perception of the relative brightness of colour. In Experiment 1 in which the seal responded to the relative brightness of blue and green instead of colour, we found that brightness perception was mediated by the rods. Experiment 2 showed that the spectral sensitivity of another harbour seal peaks between 510 and 469 nm. It is crucial to mention that our predictions of the brightness matches do not take the absorption of the ocular media into account. We had the opportunity to measure the ocular transmittance function of one juvenile harbour seal (see online resource 1) and found that the wavelength-specific absorption of the ocular media shifts the sensitivity peak of the rods from 496 to 505 nm. This would explain our findings and supports our hypothesis that vision in the harbour seal is purely scotopic at an ambient and stimulus luminance of 0.5 and 2 cd/m^2^, respectively. These light levels would lead to mesopic vision in other mammals including humans (Hammod and James 1971; Roth et al. 2008; Virsu et al. 1987).

The hypothesis that the harbour seal as well as the California sea lion have purely scotopic vision even in bright light is supported by flicker photometric ERG investigations that failed to find any cone contribution to the spectral sensitivity in the harbour seal even at an ambient illumination of 495 lx (Levenson et al. 2006). The same ERG procedures have been successfully employed to access cone-generated signals in a wide range of terrestrial mammals (Jacobs 1993) and even in the owl monkey, a nocturnal species with a similar low cone ratio in its retina (Jacobs et al. 1993) (1–2 % of all photoreceptors in the central retina of *Phoca* are cones, Peichl and Moutairou 1998; and in the owl monkey, the proportion of cones is 2 %, Wikler and Raric 1990). It is therefore possible that the secondarily aquatic lifestyle of seals has led to the evolution of visual information processing that makes them functional rod monochromats at all light levels. To confirm this, increment spectral sensitivity functions at different light levels should be obtained and more data on the ocular transmittance of seals are needed.

If cones are functional in harbour seals, they do not mediate colour vision. As only 1–2 % of the photoreceptors in the central retina of seals are cones, the question arises in which way and how much this small proportion of cones can contribute to the sense of sight in seals. In other words: Why do harbour seals have cones?

## Electronic supplementary material

Below is the link to the electronic supplementary material.

Supplementary material 1 (PDF 1581 kb)
