# Supplementary material for: Why do seals have cones? Behavioural evidence for colour-blindness in harbour seals
Source: Anim Cogn. 2014 Dec 2;18(2):551–60. doi: 10.1007/s10071-014-0823-3 (PMC4320766; doi:10.1007/s10071-014-0823-3)
Supplement: Supplementary file 1 — Supplementary material 1 (PDF 1581 kb) [file 10071_2014_823_MOESM1_ESM.pdf]

Why do seals have cones? Behavioural evidence for colour-blindness in harbour seals.

Christine Scholtyssek[1, 2, *], Almut Kelber[1], Guido Dehnhardt[2]

[1]Lund Vision Group, Functional Zoology, Department of Biology, Lund University, Sölvegatan 35, SE22362 Lund, Sweden

[2]Sensory and Cognitive Ecology, Institute for Biosciences, Rostock University, Albert-Einstein-Str. 3, D-18059 Rostock, Germany

email: Christine.Scholtyssek@biol.lu.se

Figures of the experimental apparatus used for each of the experiments

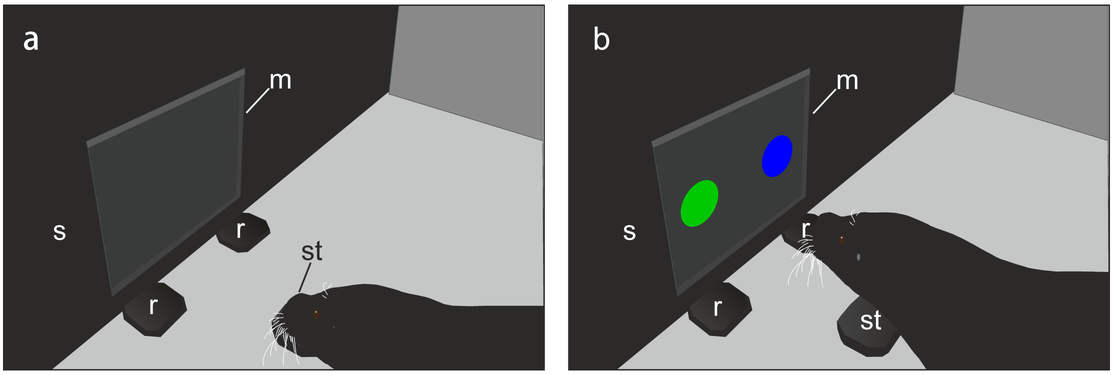

Figure ESM1: The harbour seal Nick performing the two choice procedure of Experiment 1. A black polyethylene screen **s** served for the experimenter to hide from the seal's view. a) Before a trial Nick rested in front of the monitor **m** by touching a stationing target **st** with its muzzle. b) As soon as the stimuli appeared on the monitor, Nick was trained to choose one of them by touching the corresponding response target **r** with its muzzle.

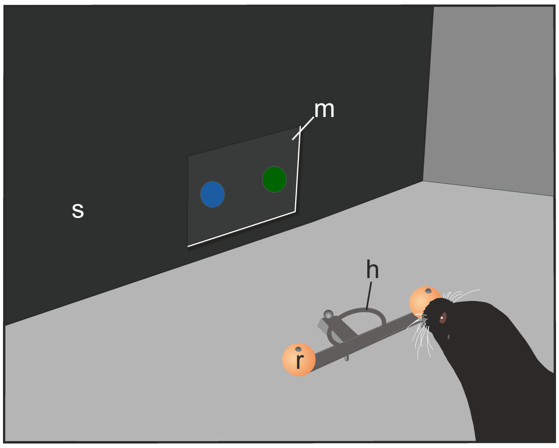

Figure ESM2: The harbor seal Luca performing the brightness discrimination task of Experiment 2. Luca stationed by placing his head in a hoop station **h** that was installed 50 cm from the center of the monitor **m**. When a stimulus pair was presented on the monitor, the seal pulled its head from the hoop station and indicated the position of the brighter colour by touching one of two response targets **r** with its muzzle.

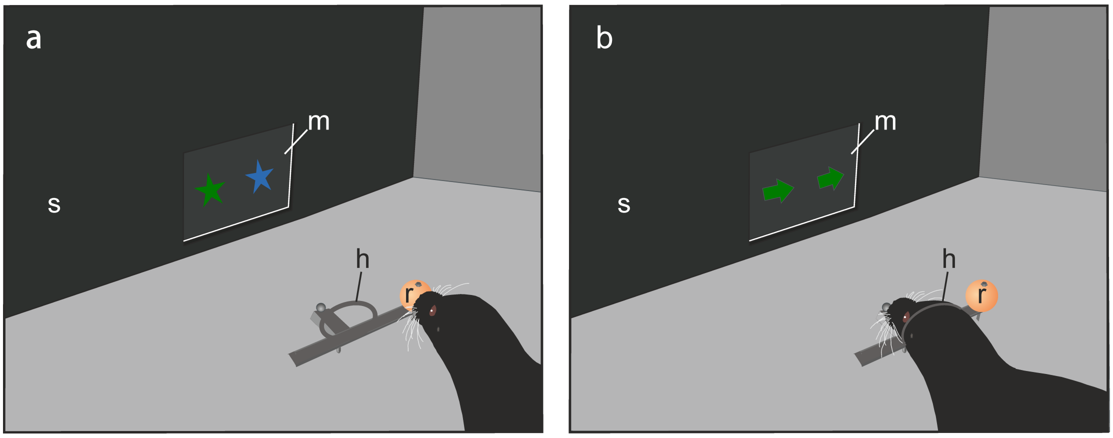

Figure ESM3: The harbour seal Luca performing the same/different task of Experiment 2. a) “Different” response: The seal was required to indicate "different" by touching the response target **r** with its muzzle. b) “Same” response. The seal was required to respond “same” by remaining in the hoop station **h** for 5 sec. after stimulus onset.

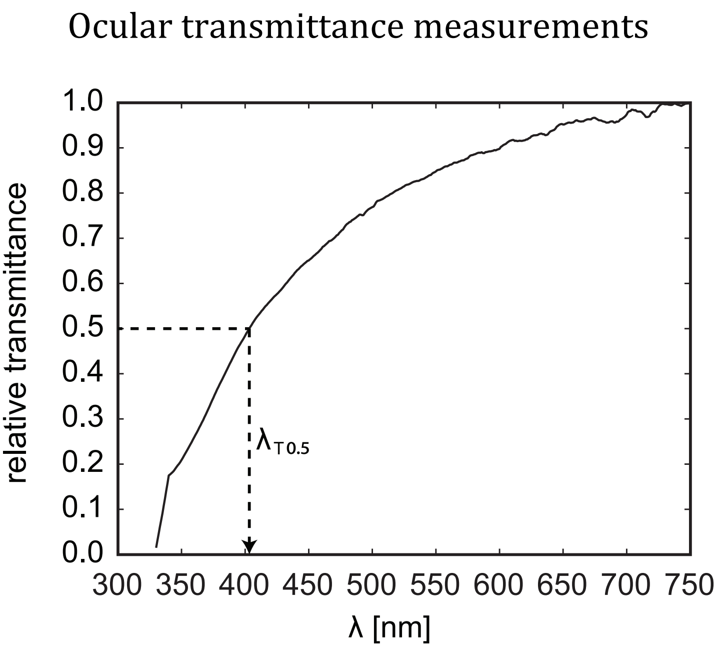

Figure ESM4: Ocular transmittance function of the eyes of a harbor seal. The curve represents the average of 4 measurements (2 left eye, 2 right eye). The wavelength of 50% transmittance ($\lambda_{T0.5}$) is 406 nm as indicated by the arrows.

Material and Methods

Eyes were obtained from a juvenile harbor seal that died during the rehabilitation program of the German province Schleswig-Holstein.

The eyes were removed 2 hours after death and measured immediately. Methods for the transmission measurements were adapted from Lind et al. (2013). For the transmission measurement, a circular window (10-15 mm wide) was cut in the back of the eye to remove sclera, choroid and retina. The eye was then placed with the lens

facing down in a custom-made matte black plastic container filled with 340mOsmol kg^{-1} phosphate-buffered saline (PBS) solution. The container had a 5mm fused silica window in its bottom and a 5mm aperture in its lid. Fused silica light guides (1000μm in diameter, Ocean Optics) were connected to these openings to send light from a Xenon light source through the eye to a spectrometer (USB2000, Ocean Optics). Reference measures were taken by measuring the transmittance of the container filled with PBS.

Transmittance measurements were processed using Spectra Suit (Ocean Optics) and Matlab (The Mathworks). The two ocular transmittance function obtained from each eye were smoothed using an 11-point running average and normalized with respect to the maximum transmittance. Subsequently all 4 measurements were averaged.

Reference

Lind O, Mitkus M, Olsson P, Kelber A. 2013 Ultraviolet sensitivity and colour vision in raptor foraging. J. Exp. Biol. 216, 1819–1826. doi:10.1242/jeb.082834
